# Theoretical Study of Absolute Entropy, Entropy of Formation, and Gibbs Energy of Formation of Two Novel Macromolecules Obtained by the Solid State

**DOI:** 10.1021/acsomega.5c02976

**Published:** 2025-06-10

**Authors:** Miguel A. García-Castro, Fausto Díaz-Sánchez, Maura Cárdenas-García, Jesús A. Arzola-Flores, Vladimir Carranza-Téllez

**Affiliations:** † Facultad de Ingeniería Química de la Benemérita Universidad Autónoma de Puebla, 18 Sur y Av. San Claudio, C.P. 72570 Puebla Pue, Mexico; ‡ Laboratorio de Fisiología Celular, Facultad de Medicina de la Benemérita Universidad Autónoma de Puebla, C.P. 72570 Puebla Pue, Mexico; § Centro de Química, Instituto de Ciencias, Benemérita Universidad Autónoma de Puebla, 72570 Puebla, Mexico

## Abstract

In this study, we present a solid-state reaction that enables the novel synthesis of diimidetricarboxylic acids (DITAs) in good yields without using harmful solvents. The synthesis route offers advantages, including satisfactory efficiency and easy product purification by sublimation. The reaction utilizes trimellitic anhydride (TMA) with 3,4- and 3,5-diaminobenzoic acid to obtain the desired DITAs. Products were characterized using differential scanning calorimetry (DSC), Fourier transform infrared spectroscopy (FTIR), proton nuclear magnetic resonance (^1^H NMR), and mass spectrometry (MS). The enthalpies of formation in the gas phase of DITAs (Δ_*f*_ *H*°) were calculated, occupying absolute entropies (*S*°), entropies of formation (Δ_*f*_ *S*°), and Gibbs energies of formation (Δ_*f*_ *G*°). These properties were determined using the hybrid method B3LYP functional and the computational technique machine learning. Our theoretical studies revealed that it is possible to predict thermochemical properties with machine learning as accurately as with density functional theory (DFT).

## Graphical abstract

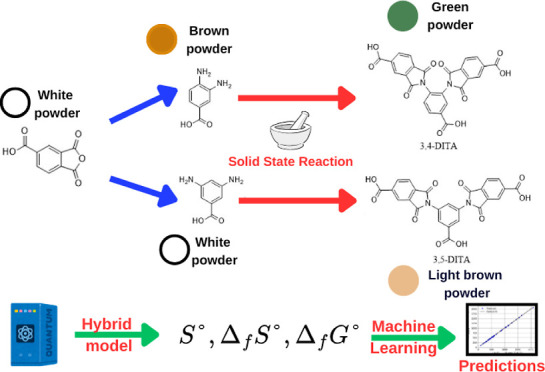

## Introduction

Solid-state reactions are efficient synthetic strategies used to obtain products with fewer polluting byproducts by fusing two starting materials in a single vessel.^1–4^ In the synthesis of diimidetricarboxylic acids, the byproduct is water in the liquid phase, which evaporates rapidly.^5^ These reactions have gained popularity in academic and industrial laboratories due to their simplicity and versatility.^6–8^ The elimination of intermediate isolation and purification steps conserves energy, time, and resources.^9^

Solid-state synthesis has become a significant field in both organic and inorganic chemistry.^10–12^ This approach enables rapid production of high-melting-point macromolecules while avoiding environmentally harmful solvents and catalysts, all while maintaining high yields.^13,14^

The resulting products serve as important precursors in polymer synthesis.^15^ They may exhibit cytotoxic effects on cancer cells due to their acidic and aromatic groups, particularly carbonyl groups in the imide group.^16,17^ Additionally, these compounds demonstrate antibacterial and fungicidal properties.^18–20^

Cyclic imides are heterocyclic compounds characterized by carbonyl groups located within the same ring.^21,22^ These compounds demonstrate chemical stability and are consequently prevalent in polymers, pharmaceuticals, and industrial materials.^23–25^ Synthetic derivatives of heterocyclic organic compounds have recently garnered scientific interest due to their diverse pharmaceutical activities. Notably, nitrogen-based compounds have shown significant efficacy against fungi, bacteria, and cancer.^26,27^

When introducing novel molecules in industrial, medical, or pharmaceutical domains, understanding stability, reactivity, and thermochemical properties is crucial for risk mitigation.^28^ Contemporary computational chemistry programs employing Gn^29,30^ or CBS^31^ methods provide high-accuracy predictions compared to experimental values. However, these methodologies require sophisticated software and extensive computational time, particularly for macromolecular systems.

Alternative estimation methods based on functional group contributions (group additivity values (GAVs)), such as those proposed by Benson,^32,33^ Gani,^34,35^ and Naef,^36–38^ which are based on the proposition that the thermodynamic properties of a molecule can be derived from the sum of its functional groups, i.e., how the atoms in a molecule are bonded. Nevertheless, these approaches are constrained by limited functional group availability for novel molecular structures.

Addressing these challenges, our research group has explored machine learning (ML) for property prediction through the GAV methodology.^39,40^ Utilizing regression or neural network methods^41^ enables obtaining results that closely approximate experimental values while significantly reducing computational resources and time compared to conventional computational chemistry programs. It also allows the functional groups for certain families of compounds to be updated, and the value of some GAVs not reported to be obtained.

In this study, two DITAs were synthesized in the solid state without solvent utilization (Figure 1). Their fusion temperatures were determined via differential scanning calorimetry (DSC), and structures were characterized through infrared spectroscopy (FTIR), proton nuclear magnetic resonance (^1^H NMR), and mass spectrometry (MS).

**Figure 1:**
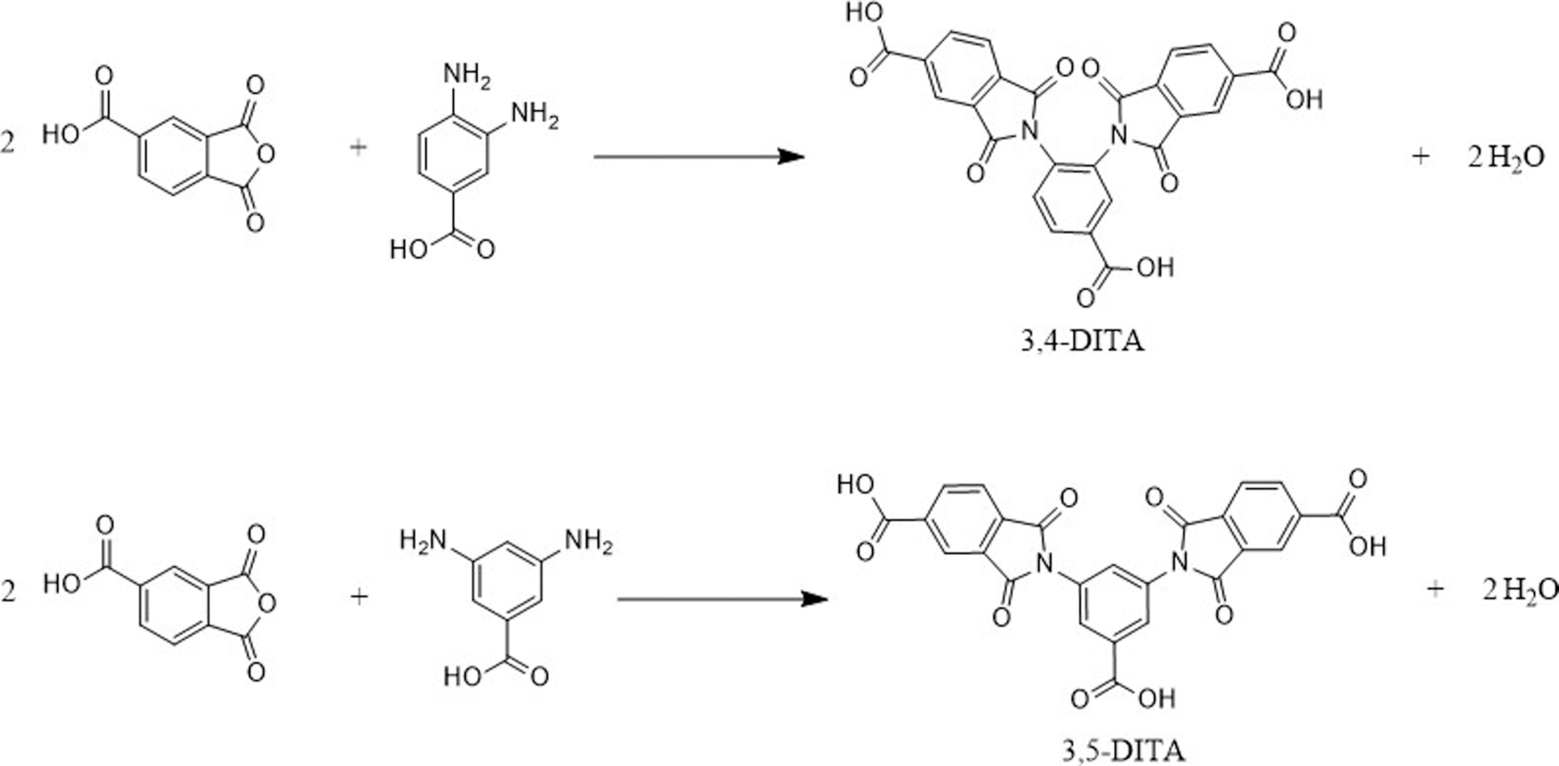
Reaction synthesis of 3,4-DITA and 3,5-DITA.

In addition, a data set of 77 values for the absolute entropy was compiled and theoretically obtained using the B3LYP/6-311++G­(d,p) method. B3LYP, a hybrid functional within density functional theory (DFT), was employed to optimize the geometry and calculate the properties of the interest. This functional combines Hartree–Fock (HF) exchange with Becke’s exchange functional (B3) and the correlation functional developed by Lee, Yang, and Parr (LYP). The 6-311++G­(d,p) atomic basis set was utilized to ensure accurate results, particularly for small- and medium-sized molecules. This combination of B3LYP and 6-311++G­(d,p) is widely recognized for its ability to balance computational cost and accuracy in calculations of geometries, energies, and molecular frequencies. The methodology has been tested in previous studies, demonstrating its efficiency and reliability in predicting thermodynamic properties, thus reducing the computational time and resources required for such calculations.^42^

In addition, the Gibbs energy was obtained from the experimental enthalpy of formation in the gas phase of the 77 compounds and the results of the DFT functional; the compounds considered in the data set are like those synthesized. To predict these properties, multiple linear regression (MLR) was used with the holdout (70/30) evaluation technique without replacement, an approach that has shown good results in predicting properties. The performance of the model was evaluated using the determination coefficient (*R* ^2^), root mean squared error (RMSE), and mean absolute error (MAE) as metrics to assess the accuracy and reliability of the predictions.^43,44^

The present study aims to develop a novel methodology for evaluating theoretical enthalpy of formation for carboxylic acids and imides, generating new absolute entropy (*S*°), entropy of formation (Δ_*f*_ *S*°), and Gibbs energy of formation (Δ_*f*_ *G*°) values. This methodology builds upon the group additivity values (GAVs) proposed by Benson, incorporating novel correction factors for aromaticity and structural isomerism.

## Experimental Procedure

### Materials and Instruments

Reagents were obtained from Sigma-Aldrich with the following specifications:

- 1,2,4-Benzenetricarboxylic anhydride [TMA, CAS 552-30-7]: mass fraction 0.97
- 3,4-Diaminobenzoic acid [3,4-DABA, CAS 619-05-6]: mass fraction 0.97
- 3,5-Diaminobenzoic acid [3,5-DABA, CAS 535-87-5]: mass fraction 0.98

The TMA was dried at 373.15 K under vacuum (500 mmHg) for 8 h to maintain its anhydride form. Diamines were used in the synthesis without additional purification. Instrumental analyses were conducted using the following equipment:

- Infrared spectroscopy: PerkinElmer ATR-FTIR spectrometer (4 cm^–1^ resolution).
- Proton nuclear magnetic resonance: Bruker 500 MHz spectrometer (DMSO-*d*_6_ as solvent).
- Mass Spectrometry: JEOL MStation JMS-700 spectrometer.
- Differential Scanning Calorimetry: Mettler Toledo DSC 1 (for purity tests and fusion temperature determination).

### Synthesis of Diimidetricarboxylic Acids, DITAs

DITAs were synthesized via solid-state synthesis without solvents using the following quantities: 1,2,4-benzenetricarboxylic anhydride (TMA) 0.7920 g, 3,4-diaminobenzoic acid (3,4-DABA) 0.3135 g, and 3,5-diaminobenzoic acid (3,5-DABA) 0.3103 g.

Preliminary fusion tests revealed that TMA remains stable during heating and cooling processes, whereas diamines tend to decompose.^45^ Consequently, the synthesis protocol developed by our research group involved heating TMA to its melting point (433.15 K) and immediately incorporating either 3,4-DABA or 3,5-DABA under vigorous agitation for approximately 15 min.

The resulting products were washed thrice with deionized water and dried at 473.15 K under 20 cmHg vacuum for 4 h. The synthesis yielded 3,4-DITA: a greenish compound (85% yield) and 3,5-DITA: a brown compound (92% yield).

Compounds were comprehensively characterized by FTIR, ^1^H NMR, and MS (Supporting Information, Figures S1–S8).

#### 3,4-DITA

FTIR (cm^–1^). Carboxylic acid: 3500–3580 (νOH), 1740–1800 (νC=O), 1280–1380 (δOH), 1075–1190 (νC–O). Imide 1735–1790 (νC=O), 1680–1745 (νC=O), and 1100 (νC–N–C). ^1^H NMR (500 MHz, DMSO-*d*_6_): δ­(ppm): 13.38 (s, 3H), 8.31 (dd, *J* = 4.8, 1.3 Hz, 1H), 8.27–8.17 (m, 4H), 8.05 (t, *J* = 7.5 Hz, 2H), 7.83 (t, *J* = 7.7 Hz, 2H). HRMS (FAB^+^) *m*/*z* calcd for [C_25_H_12_N_2_O_10_]^+^, 500.2345; found 500.2338.

#### 3,5-DITA

FTIR (cm^–1^). Carboxylic acid: 3500–3580 (νOH), 1740–1800 (νC=O), 1280–1380 (δOH), 1075–1190 (νC–O). Imide 1735–1790 (νC=O), 1680–1745 (νC=O), 1360 (νC–N), and 1100 (νC–N–C). ^1^H NMR (500 MHz, DMSO-*d*_6_): δ­(ppm): 13.57 (s, 3H) 8.44 (d, *J* = 6.3 Hz, 2H), 8.34 (s, 2H), 8.18 (d, *J* = 1.9, 2H), 8.12 (d, *J* = 7.7, 2H), 7.90 (t, *J* = 2.0 Hz, 1H). HRMS (FAB^+^) *m*/*z* calcd for [C_25_H_12_N_2_O_10_]^+^, 500.2345; found 500.2356.

Fusion temperatures were determined using a Mettler Toledo DSC1 instrument, precalibrated with indium for temperature and heat flow. The heating rate was 3.0 K min^–1^ using a nitrogen flow rate of 70.0 cm^3^ min^–1^ from 298.15 to 613.15 K for 3,4-DITA and from 298.15 to 573.15 K for 3,5-DITA.

### Computational Procedure

In previous research,^44^ a methodology was proposed to obtain the enthalpy of formation, both in gas and crystalline phases, for different families of compounds using the computational technique of machine learning (ML) and the functional group contribution methods proposed by Benson.^46^ Since this methodology has provided good estimates, it was proposed to perform a similar methodology for the prediction of absolute entropy (*S*°), entropy (Δ_*f*_ *S*°), and Gibbs energy of formation (Δ_*f*_ *G*°), since these values are rarely reported experimentally, and it was chosen to perform calculations by DFT.

The molecular calculations were performed at the B3LYP/6-311++G­(d,p) theoretical level to estimate the gaseous-phase heat capacities of DITAs.^47^ This functional and basis set had been previously validated for anhydrides,^42^ yielding good approximations of experimental values for both vibrational frequencies and heat capacities. The obtained geometries were characterized as true minimums after calculating vibrational frequencies at the same theoretical level. Calculations were executed using the Gaussian 09 computer code.^48^

A data set of 77 organic molecules (comprising carboxylic acids and imides, which are the basis of the structure of DITAs) was generated with previously reported enthalpies of formation in the gas phase (Δ_*f*_ *H*°) and absolute entropies (*S*°) calculated at the B3LYP theoretical level. These properties were used to determine entropies of formation (Δ_*f*_ *S*°) and Gibbs energies of formation (Δ_*f*_ *G*°) using Hess’s law (eq 1) and eq 2.

$$mC\left(s\right)+\frac{n}{2}H_{2}\left(g\right)+\frac{p}{2}O_{2}\left(g\right)+\frac{o}{2}N_{2}\left(g\right)=C_{m}H_{n}O_{p}N_{o}\left(g\right)$$ 1

2 $$\Delta_{f}G{{}^{\circ}}_{m}\left(g\right)=\Delta_{f}H{{}^{\circ}}_{m}\left(g\right)-T\Delta_{f}S{{}^{\circ}}_{m}\left(g\right)$$

The absolute entropy values were as follows: *C*(*s*) of (5.74 ± 0.10) J mol^–1^ K^–1^, H_2_(g) of (130.571 ± 0.003) J mol^–1^ K^–1^, O_2_(*s*) of (205.043 ± 0.005) J mol^–1^ K^–1^, and N_2_(*s*) of (191.500 ± 0.004) J mol^–1^ K^–1^.^49^

After data set completion, the thermochemical properties of DITAs were predicted using machine learning via multiple linear regression (MLR) with Python and the scikit-learn library.^50^ The code used in this work, as well as the data set, can be found in the following GitHub repository: https://github.com/FDS116/DITAs.

## Results and Discussion

### Synthesis and Characterization

Several reaction parameters of bis­(phthalimide)­s have been determined from conventional synthesis methods, such as the type of solvents and condensation agents,^16,18,51^ and from mechanochemistry with and without the use of catalysts.^13,22,52^ On the other hand, synthesis routes based on the order of addition of reagents have also been tested to obtain a preferential distribution of arrangements in the recurrent unit during the formation of poly­(amida-mimida)­s.^53,54^ Then, this research exposes the first approach of an ecofriendly reaction to obtain DITAs without the use of solvents, based on reaching the melting point of one of the reagents and where the purification method was carried out by sublimation, achieving yields greater than 85%.

FTIR characterization of both structural isomers revealed nearly identical absorption bands. The acid group displayed a band at 1790 cm^–1^ (νC=O, carbonyl), while the imide group showed bands at 1735 cm^–1^ (νC=O, carbonyl) and 1100 cm^–1^ (νC–N–C). Notably, 3,5-DITA exhibited an additional signal at 1360 cm^–1^ (νC–N), as illustrated in Supporting Information Figure S9. Bands corresponding to 1,3,4- and 1,3,5-trisubstituted benzene ring vibrations were between 2000 cm^–1^ and 1600 cm^–1^.

The ^1^H NMR spectra of DITAs, performed in DMSO-*d*_6_, shown in Supporting Information Figures S3 and S4, revealed distinct chemical shifts. The 3,4-DITA spectrum displayed five signals at δ = 13.38, 8.31, 8.27–8.17, 8.05, and 7.83 ppm, while the 3,5-DITA spectrum showed six signals at δ = 13.57, 8.44, 8.34, 8.18, 8.12, and 7.90 ppm, respectively. Both DITAs exhibited 12 proton integrations corresponding to carboxylic acid and aromatic ring protons. For 3,4-DITA, the signal at δ = 8.31 ppm was assigned to the proton at position two of the trisubstituted aromatic ring, whereas for 3,5-DITA, the corresponding signal appeared at δ = 7.90 ppm at position six.

In the case of MS characterization (Figures S5 and S8 in the Supporting Information), the signal with the highest relative abundance (base peak) for both DITAs was 411 *m*/*z*. The molecular ion peak (*m*/*z* = 500) corresponds to the molecular weight of the compound DITA, and it was identified from fast atom bombarding ionization mode (FAB^+^).

The fusion temperatures of 566.87 K for 3,4-DITA and 546.15 K for 3,5-DITA were determined by DSC (Supporting Information Figures S10 and S11). In both cases, vaporization signals appeared above 596.76 K, indicating the thermal stability of these compounds in the liquid state.

### Theoretical Results

Absolute entropies were obtained from structures optimized by using the B3LYP hybrid method for both synthesized compounds and the reference data set. A third-order polynomial fit (*R* ^2^ = 0.9999) of *C*_p,m_(g) vs *T* was derived from the determined vibrational frequencies (eqs 3–6 and Tables S1 and S2 in Supporting Information). These properties were used to calculate the equilibrium constants for each synthesis reaction and the entropies of formation of the data set.

$$C_{p,m}\left(3,4‐DABA,g\right)/(J\;mol^{-1}{\;K}^{-1})=-2.29\times 10^{-7}{\left(\frac{T}{K}\right)}^{3}-1.54\times 10^{-4}{\left(T/K\right)}^{2}+0.63\left(T/K\right)+0.59$$ 3

4 $$C_{p,m}\left(3,5‐DABA,g\right)/(J\;mol^{-1}{\;K}^{-1})=-1.11\times 10^{-7}{\left(\frac{T}{K}\right)}^{3}-2.89\times 10^{-4}{\left(T/K\right)}^{2}+0.68\left(T/K\right)+1.10$$

$$C_{p,m}\left(3,4‐DITA,g\right)/(J\;mol^{-1}{\;K}^{-1})=2.36\times 10^{-7}{\left(\frac{T}{K}\right)}^{3}-0.0014\times 10^{-4}{\left(T/K\right)}^{2}+2.16\left(T/K\right)-40.24$$ 5

$$C_{p,m}\left(3,5‐DITA,g\right)/(J\;mol^{-1}{\;K}^{-1})=2.28\times 10^{-7}{\left(\frac{T}{K}\right)}^{3}-0.0014\times 10^{-4}{\left(T/K\right)}^{2}+2.15\left(T/K\right)-38.07$$ 6

Regarding Gibbs formation energies, the data set was constructed from previously reported enthalpies of formation for 77 organic molecules and the formation entropies determined in this investigation using eq 2. New absolute entropy (*S*°), entropy of formation (Δ_*f*_ *S*°), and Gibbs energy of formation (Δ_*f*_ *G*°) values were generated for Bensońs group additivity values (GAVs) (Figures 2,3,4). Structural isomerism and aromaticity corrections were applied to improve predictions for the compounds of interest. Table 1 presents the evaluation metrics calculated using the holdout (70/30) technique without replacements with a fixed seed to ensure reproducible results.

**Figure 2:**
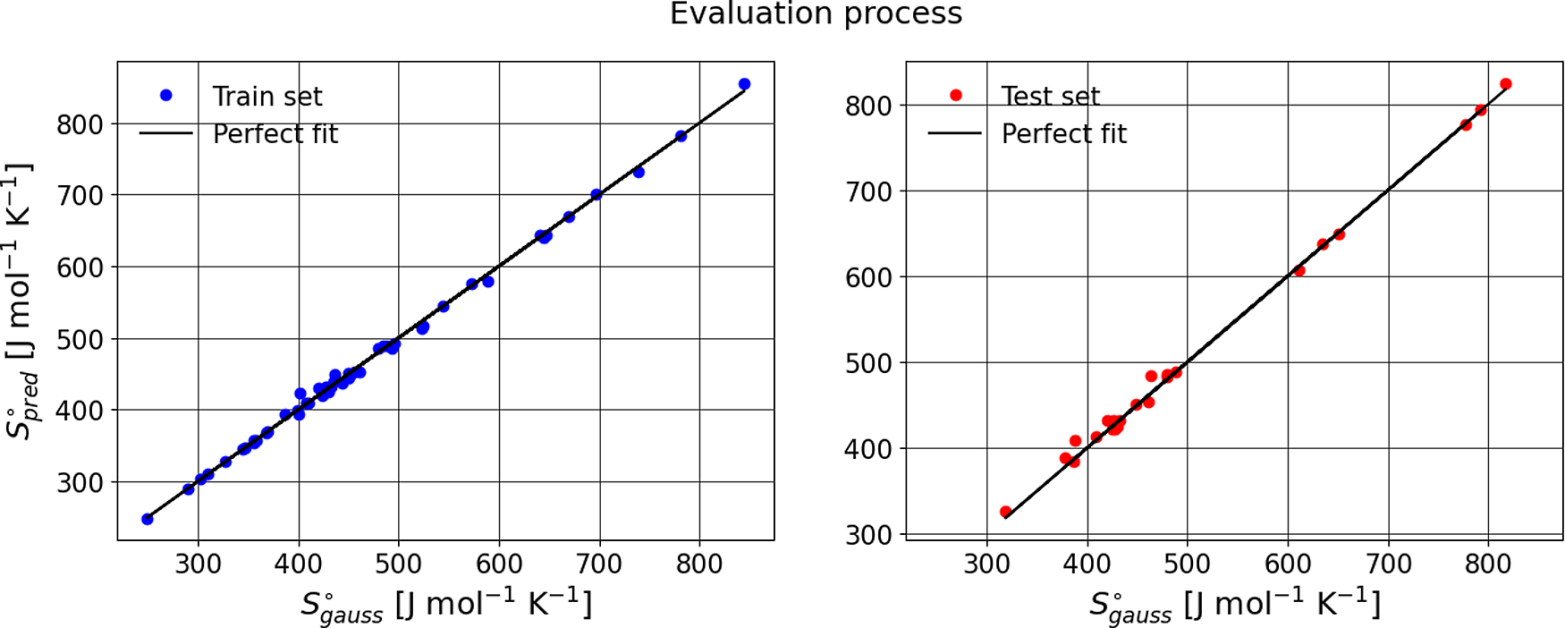
Predicting training and test sets for absolute entropy.

**Figure 3:**
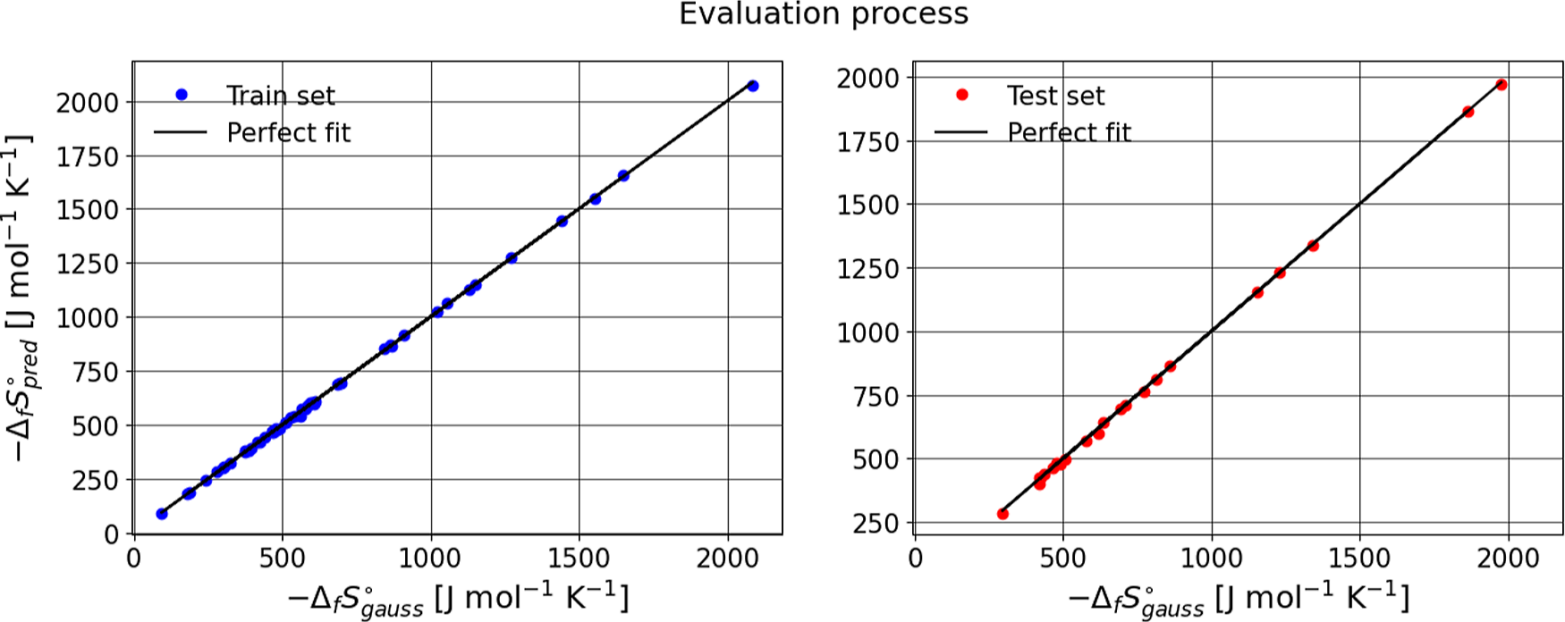
Predicting training and test sets for formation entropy.

**Figure 4:**
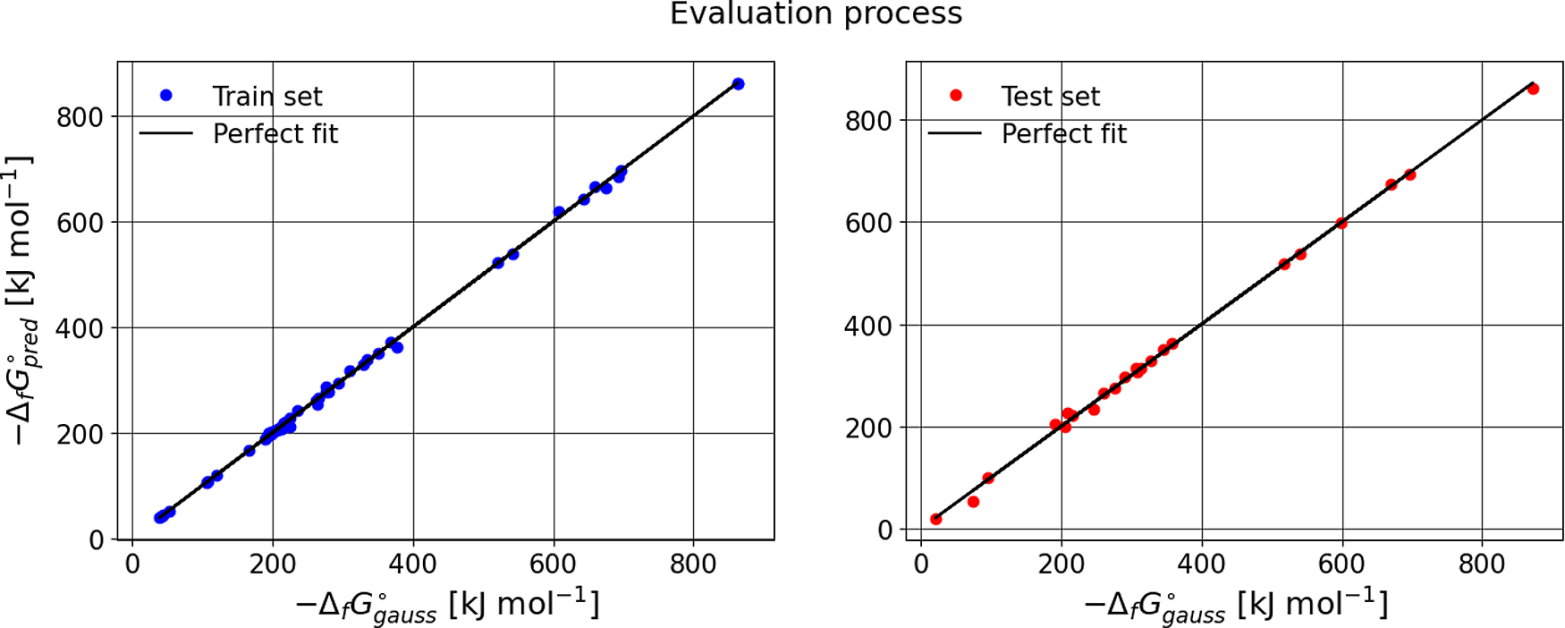
Training and test set prediction for absolute Gibbs energy.

**Table 1:** Evaluation Metrics for Linear Regression

*S*° (g, 298.15 K)								
	train	test		train	test		train	test
*R* ^2^	0.9979	0.9964	MAE	3.9272	6.1227	RMSE	5.7921	8.247
-Δ_*f*_ *S*° (g, 298.15 K)								
	train	test		train	test		train	test
*R* ^2^	0.9998	0.9996	MAE	3.9272	6.3722	RMSE	5.7921	8.481
-Δ_*f*_ *G*° (g, 298.15 K)								
	train	test		train	test		train	test
*R* ^2^	0.9994	0.9986	MAE	2.9398	5.7992	RMSE	4.9202	7.8588

In general, the evaluation metrics for the three cases indicate that the model performs well on the training set, with an *R* ^2^ close to 1, reflecting excellent predictive ability. However, when the model is evaluated on the test set, a slight decrease in *R* ^2^ and an increase in MAE and RMSE are observed, suggesting a small loss of precision in generalization. Despite these changes, the difference is not significant, implying that the model still generalizes reasonably well and maintains a good balance between fit and predictive capability.

The absolute entropy values for 3,4-DITA and 3,5-DITA calculated at the B3LYP/6-311++G­(d,p) theoretical level were 836.9 and 846.0 J mol^–1^ K^–1^, respectively. Their entropies of formation were −1306.7 and −1297.6 J mol^–1^ K^–1^, respectively.

Table 2 presents the machine learning (ML)-predicted results and their deviations from the theoretical calculations (shown in parentheses). These results validate the model’s predictive capability.

**Table 2:** Absolute Entropies, Entropies of Formation, and Gibbs Energies of Formation for the Compounds at 298.15 K and 0.1 MPa Were Obtained with ML

compound	*S*° (g)/J mol^–1^ K^–1^	Δ_*f*_ *S*° (g)/J mol^–1^ K^–1^	Δ_*f*_ *G*° (g)/kJ mol^–1^
3,4-DITA	839.8 (−2.9)	–1303.8 (−2.9)	–1017.9
3,5-DITA	848.0 (−2.0)	–1295.6 (−2.0)	–1023.8

Using ML-predicted Gibbs energies of formation, the gas-phase enthalpies of formation were determined as −1406.6 kJ mol^–1^ and −1410.1 kJ mol^–1^ for 3,4- and 3,5-DITA, respectively. The enthalpy of formation was estimated using the absolute entropy obtained by the B3LYP/6-311++G­(d,p) theoretical level and the Gibbs energy from the ML, Table 3.

**Table 3:** Comparison of Standard Molar Enthalpies of Formation at 298.15 K and 0.1 MPa in kJ mol^–1^

compound	Δ_*f*_ *H*° (g)^a^	Δ_*f*_ *H*° (g)^b^	Δ
3,4-DITA	–1406.6	–1407.5	–0.9
3,5-DITA	–1410.1	–1410.7	–0.6

a Value obtained by ML.

b Value obtained by B3LYP and ML.

The ML-derived enthalpy values closely match theoretical calculations, with the primary difference being computational time (Gaussian calculations required several hours). The enthalpy of isomerization (Δ_iso_ *H*°) between the compounds is -(3.4 ± 1.1) kJ mol^–1^.

Given that Gibbs energy serves as a stability criterion, the 3,5-DITA isomer is more stable than 3,4-DITA. This stability difference stems from the steric hindrance generated by the carbonyls of the imide groups in the meta position of 3,4-DITA (Figure 5).

**Figure 5:**
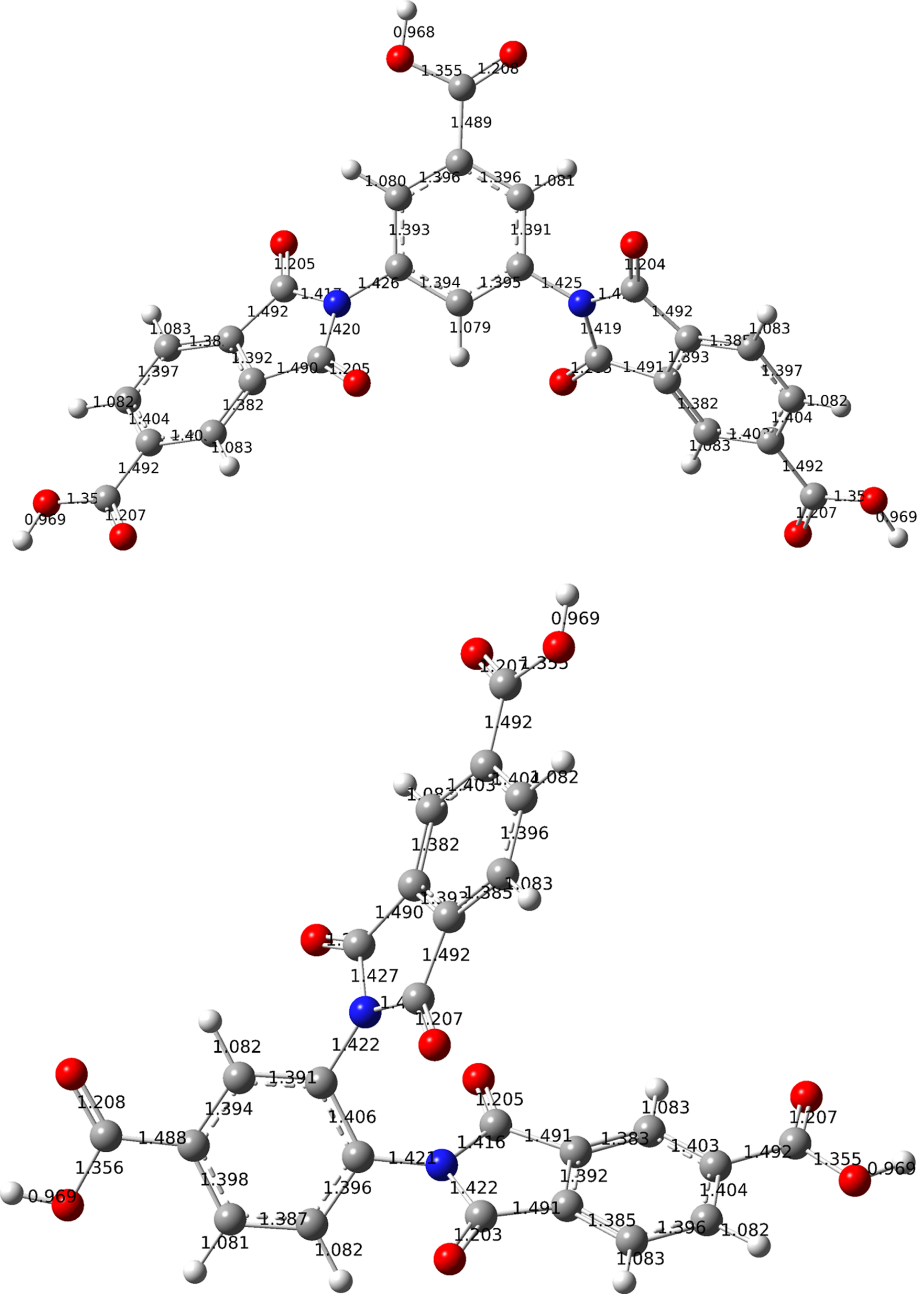
Optimized structures with B3LYP/6-311++G­(d,p) for 3,4-DITA (left) and 3,5-DITA (right). Bond lengths in nm are included.

An advantage of performing the GC method by MLR is the ability to obtain regression coefficients for manual estimation. This approach allows updating the values proposed by the Benson method for the gas phase properties, specifically for the carboxylic acid family and imide compounds (R–CO–N–CO–R′), as detailed in Table 4. Similarly, Table S3 provides a manual calculation procedure for the synthesized compounds.

**Table 4:** Values for Benson Groups in the Gas Phase Obtained by MLR

Group	*S*°	Δ_*f*_ *S*°	Δ_*f*_ *G*°
	J mol^–1^ K^–1^	J mol^–1^ K^–1^	kJ mol^–1^
CO-(O)(CO)	9.79	–28.16	–79.72
CO-(C_D_)(O)	–0.17	6.5	–20.23
CO-(C)(O)	13.61	–24.34	–79.44
CO-(H)(O)	–32.67	77.16	–181.71
CO-(O)(C_B_)	18.43	–47.22	23.22
O-(H)(CO)	8.98	–16.06	–337.89
O-(C)(C_B_)	18.77	–60.84	–37.25
C_D_-(H)(CO)	21.74	–23.82	–67.02
C_B_-(CO)(C_B_)_2_	41.55	–78.55	–21.67
C_B_-(O)(C_B_)_2_	18.77	–60.84	–37.25
C-(CO)(C)_3_	12.26	–74.12	55.26
C-(H)_2_(CO)(C)	37.04	–99.27	–9.41
C-(H)_3_(O)	18.77	–60.84	–37.25
C-(H)_3_(C)	–5.39	6.09	–114.10
C-(H)_3_(C_B_)	3.46	7.28	–48.20
C-(H)_2_(C)_2_	31.12	–105.19	10.78
C_D_-(H)_2_	–0.17	6.5	–20.23
C_B_-(H)(C_B_)_2_	2.38	2.38	–8.79
C_B_-(C)(C_B_)_2_	37.91	–102.23	31.24
C-(H)_2_(C)(C_*B*_)	34.45	–109.51	79.44
C-(C)_4_	0.00	0.00	25.81
CH_3_(qua)	24.52	–148.24	110.51
C_*B*_-(C_B_)_3_	18.22	–58.55	40.84
CO-(C)(N)	12.21	–14.54	–153.75
N-(H)(CO)_2_	–23.65	78.45	–124.97
C-(H)_3_(N)	–3.66	34.20	–75.91
N-(CO)_2_(C)	21.61	–50.46	–36.18
CO-(C_D_)(N)	21.91	–30.32	–46.79
CO-(N)(C_B_)	23.12	–31.33	–44.89
CB-(NO_2_)(C_B_)_2_	58.75	–176.76	57.45
C-(H)_2_(N)(C)	25.27	–84.66	39.73
C_B_-(N)(C_B_)_2_	30.65	–66.09	38.44
N-(CO)_2_(C_B_)	30.65	–66.09	38.44
rsc	–20.22	–20.22	4.19
radical 1	2.78	2.78	–4.35
radical 2	0.32	0.32	–3.34
radical 3	–3.47	–3.47	–2.00
radical 4	0.86	0.86	–1.81
correction o-	–3.56	–3.56	29.33
correction m-	–1.91	–1.91	14.90
correction p-	–9.77	–9.77	17.47
*H*_*f*0_	272.69	–153.46	168.44

The equilibrium reaction for gas-phase synthesis of 3,4- and 3,5-DITA (Figure 1) was analyzed. To estimate the enthalpies of formation in the gas phase of 3,4- and 3,5-DABA, enthalpic differences were calculated using *o*-phenylendiamine (Δ_*f*_ *H*°(g) = 86.6 ± 1.6 kJ mol^–1^) and *m*-phenylendiamine (Δ_*f*_ *H*°(g) = 89.6 ± 1.6)^45^ relative to benzene (82.9 ± 0.9 kJ mol^–1^).^55^ Values of (3.7 ± 1.8) kJ mol^–1^ and (6.7 ± 1.8) kJ mol^–1^ were added to benzoic acid (Δ_*f*_ *H*°(g) = -295.7 ± 0.1 kJ mol^–1^),^56^ as shown in Figure 6.

**Figure 6:**
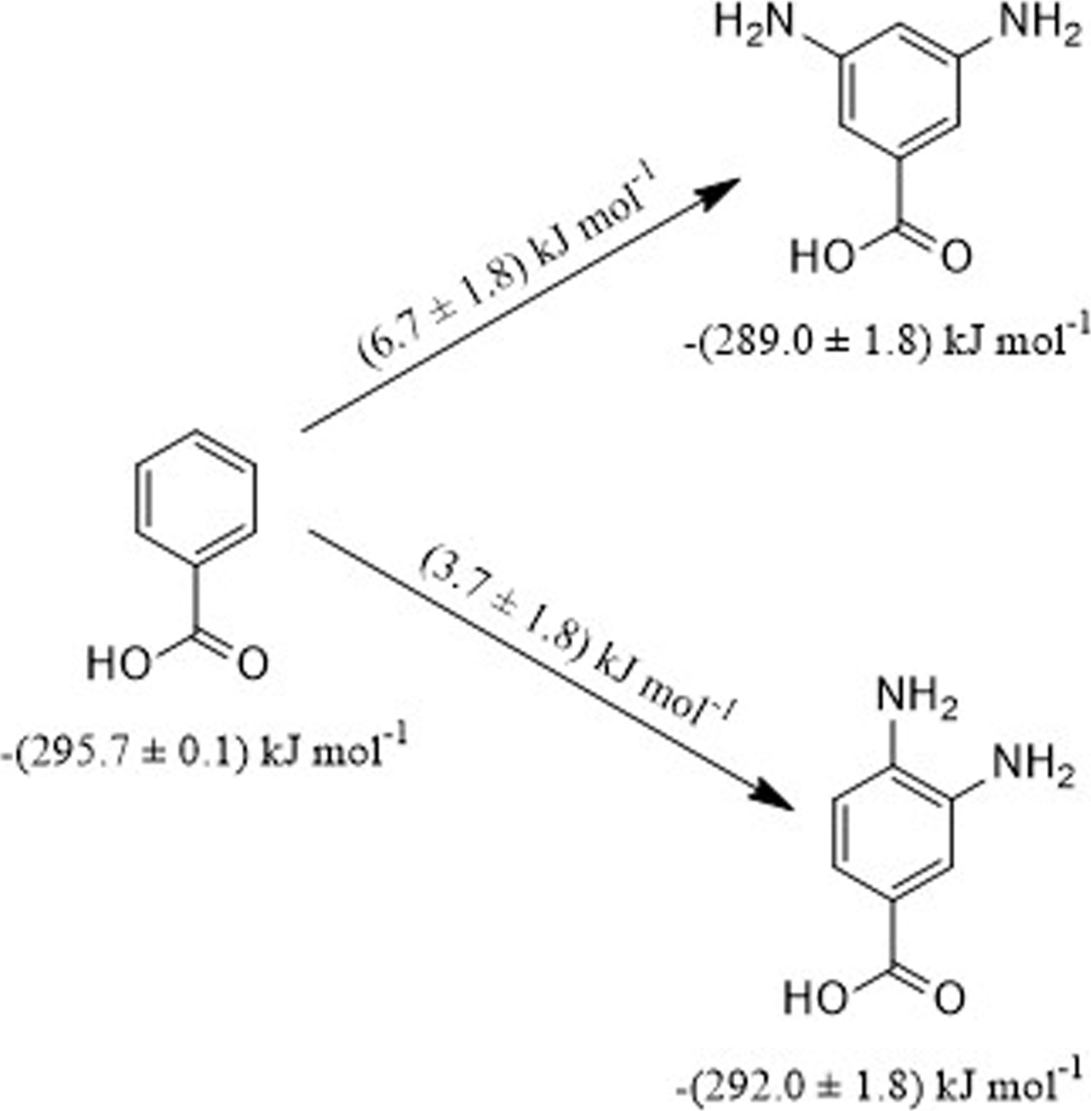
Enthalpic differences to estimate Δ_*f*_ *H*° _*m*_(g) of 3,4- and 3,5-DABA.

Following the methodology from ref 57, the equilibrium constant, standard enthalpy, standard entropy, and standard Gibbs energy of reaction for 3,4-DITA and 3,5-DITA (Figure 1) were determined in the gas phase at 298.15 K (Table 5). The following enthalpies of formation were used: Δ_*f*_ *H*°_*m*_(g): −757.7 kJ mol^–1^ for TMA,^58^ -(292.0 ± 1.8) kJ mol^–1^ for 3,4-DABA, -(289.0 ± 1.8) kJ mol^–1^ for 3,5-DABA, −1406.6 kJ mol^–1^ for 3,4-DITA, −1410.1 kJ mol-1 for 3,5-DITA, and -(241.826 ± 0.040) kJ mol^–1^ for H_2_O,^59^ along with the entropy of formation values from Table 2.

**Table 5:** Values Calculated for Reaction Synthesis of 3,4-DITA and 3,5-DITA at 298.15 K^a^

compound	Δ_*r*_ *H*°_*m*_(g)	Δ_*r*_ *S*°_*m*_(g)	Δ_*r*_ *G*°_*m*_(g)	*K*_eq_
	kJ mol^–1^	J mol^–1^ K^–1^	kJ mol^–1^	
3,4-DITA	–82.86 ± 2.06	–47.92 ± 0.17	–68.57 ± 2.06	1.03 × 10^12^
3,5-DITA	–89.36 ± 2.06	–44.72 ± 0.17	–76.03 ± 2.06	2.09 × 10^13^

a The uncertainty reported is the combined standard uncertainty.

The gas-phase synthesis of 3,4-DITA and 3,5-DITA is favored at 298.15 K and is exothermic. At the melting temperature of trimellitic anhydride (433.15 K), as shown in Tables S4 and S5 (Supporting Information), the increase in Δ_*r*_ *S*°_*m*_(g) indicates the tendency toward an irreversible reaction, with equilibrium constant (*K*°) values of 1.57 × 10^8^ and 1.36 × 10^9^ for 3,4- and 3,5-DITA, respectively.

## Conclusions

Two DITAs were synthesized in the solid state at the melting temperature of trimellitic anhydride (433.15 K), and their purification was carried out without solvents with yields of 85% and 92%, respectively. Compositional and structural characterization was performed using Fourier-transform infrared spectroscopy (FTIR), nuclear magnetic resonance (^1^H NMR), and mass spectrometry (MS). Differential scanning calorimetry (DSC) determined the melting temperature as 566.87 and 546.15 K for 3,4- and 3,5-DITA, respectively.

Group contribution (GC) models for predicting the thermochemical properties of DITAs from ML using multiple linear regression (MLR) were found to be appropriate. The enthalpy of formation was estimated by combining the absolute entropy obtained from the B3LYP/6-311++G­(d,p) level of theory with the Gibbs energy derived from ML. Ultimately, the equilibrium constant, standard enthalpy, standard entropy, and standard Gibbs energy of the gas-phase reaction were calculated.

## Supplementary Material



## Notes

The Python code to implement the ML algorithms and the data set to train, validate, and test those algorithms can be found in the GitHub repository: https://github.com/FDS116/DITAs. The data set consists of theoretically obtained values; subsequently, these values were occupied with experimental values of standard enthalpy of formation at 298.15 K; these values were taken from different references. FTIR spectra; ^1^H NMR spectra; MS spectra; fragmentation paths; comparison of FTIR spectra; DSC thermograms; results for *C*_p,m_(g) at different temperatures; estimation of thermochemistry properties; and values calculated for the reaction of 3,4- and 3,5-DITA at different temperatures (PDF)
