# Supplementary material for: Theoretical Study of Absolute Entropy, Entropy of Formation, and Gibbs Energy of Formation of Two Novel Macromolecules Obtained by the Solid State
Source: ACS Omega. 2025 Jun 10;10(24):26061–70. doi: 10.1021/acsomega.5c02976 (PMC12198991; doi:10.1021/acsomega.5c02976)
Supplement: Supplementary file 1 [file ao5c02976_si_001.pdf]

Theoretical study of absolute entropy, entropy of formation and Gibbs energy of formation of two novel macromolecules obtained by solid state

Miguel Angel García-Castro,[†] Fausto Díaz-Sánchez,[†] Maura Cárdenas-García,[‡] Jesús Andrés Arzola-Flores,[*,†] and Vladimir Carranza-Téllez[¶]

†*Facultad de Ingeniería Química de la Benemérita Universidad Autónoma de Puebla, 18 Sur y Av. San Claudio, C.P. 72570, Puebla Pue, Mexico*

‡*Laboratorio de Fisiología Celular, Facultad de Medicina de la Benemérita Universidad Autónoma de Puebla, C.P. 72570, Puebla Pue, Mexico*

¶*Centro de Química, Instituto de Ciencias de la Benemérita Universidad Autónoma de Puebla, 18 Sur y Av. San Claudio, C.P. 72570, Puebla Pue, Mexico*

E-mail: jesus.arzolaflores@correo.buap.mx

Supporting Information Available

LIST OF CONTENTS

Figure S1: FTIR spectrum of 3,4-DITA

Figure S2: FTIR spectrum of 3,5-DITA

Figure S3: ^{1}H-NMR spectrum of 3,4-DITA

Figure S4: ^{1}H-NMR spectrum of 3,5-DITA

Figure S5: MS spectrum of 3,4-DITA

Figure S6: Fragmentation path of 3,4-DITA

Figure S7: MS spectrum of 3,5-DITA

Figure S8: Fragmentation path of 3,5-DITA

Figure S9: Comparison of FTIR of DITAs

Figure S10: DSC thermogram of 3,4-DITA

Figure S11: DSC thermogram of 3,5-DITA

Table S1: Results for $C_{p,m}$(g) of 3,4- and 3,5-DABA at different temperatures

Table S2: Results for $C_{p,m}$(g) of 3,4- and 3,5-DITA at different temperatures

Table S3: Estimation of thermochemistry properties

Table S4: Values calculated for the reaction of 3,4-DITA at different temperatures

Table S5: Values calculated for the reaction of 3,5-DITA at different temperatures

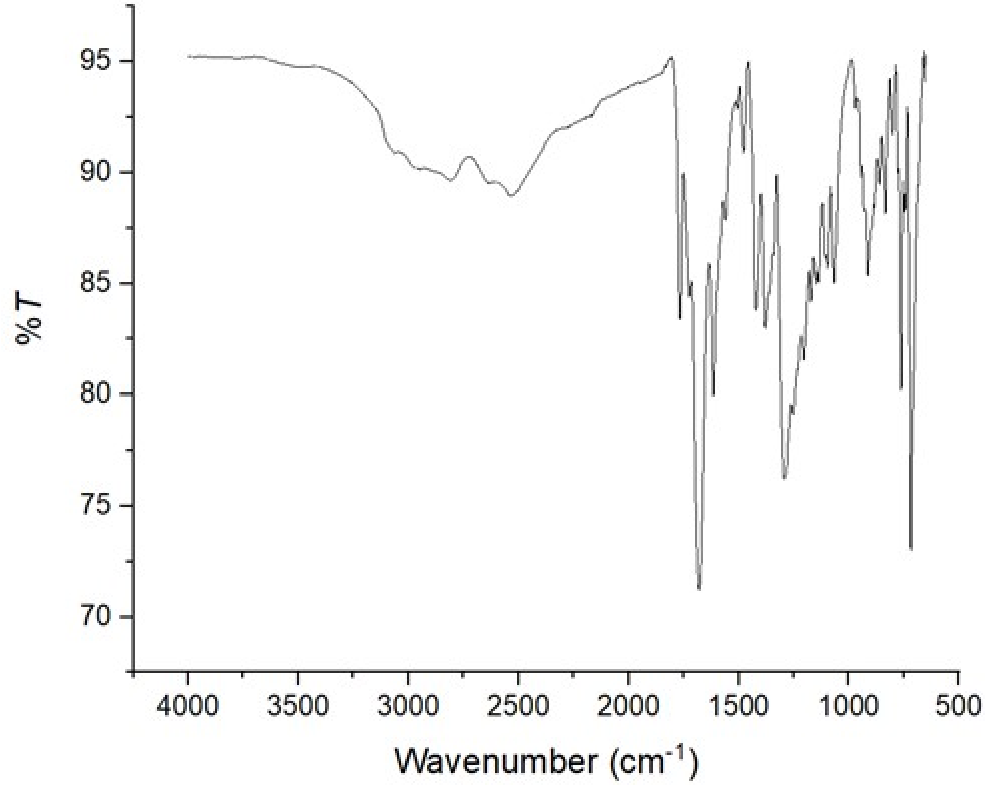

Figure S1: FTIR spectrum of 3,4-DITA.

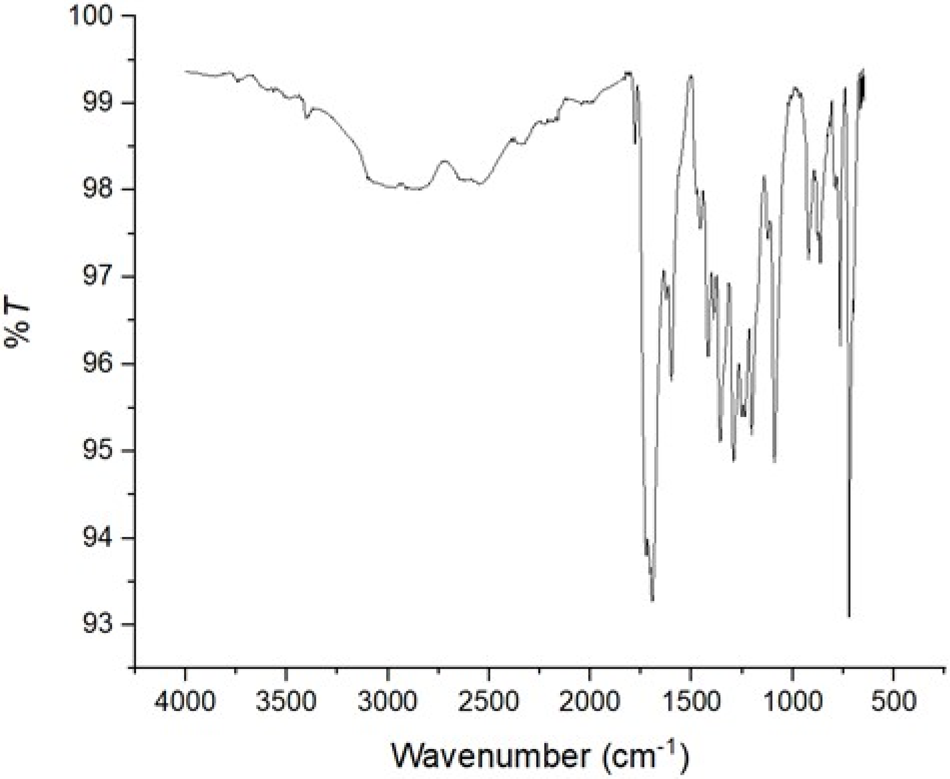

Figure S2: FTIR spectrum of 3,5-DITA.

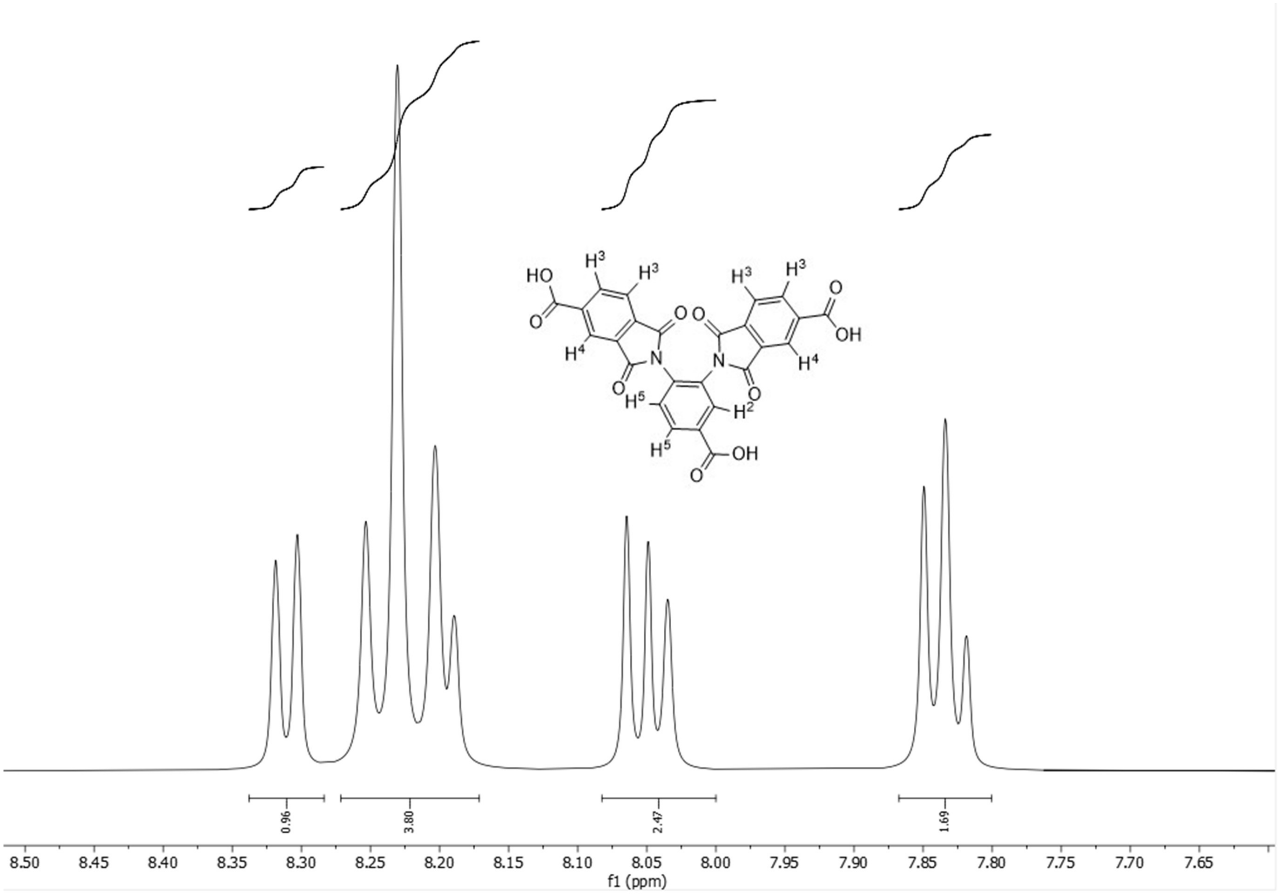

Figure S3: ^{1}H-NMR spectrum of 3,4-DITA.

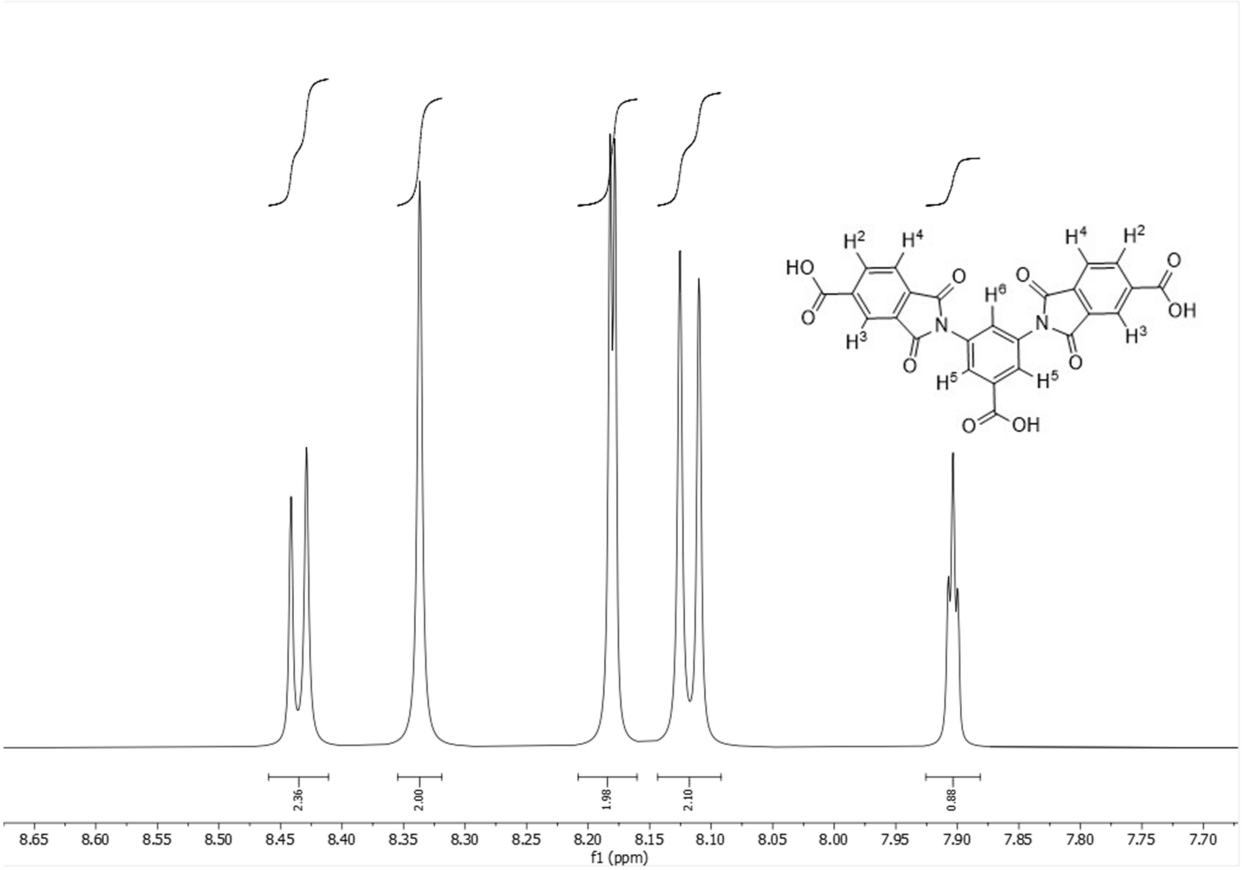

Figure S4: ^{1}H-NMR spectrum of 3,5-DITA.

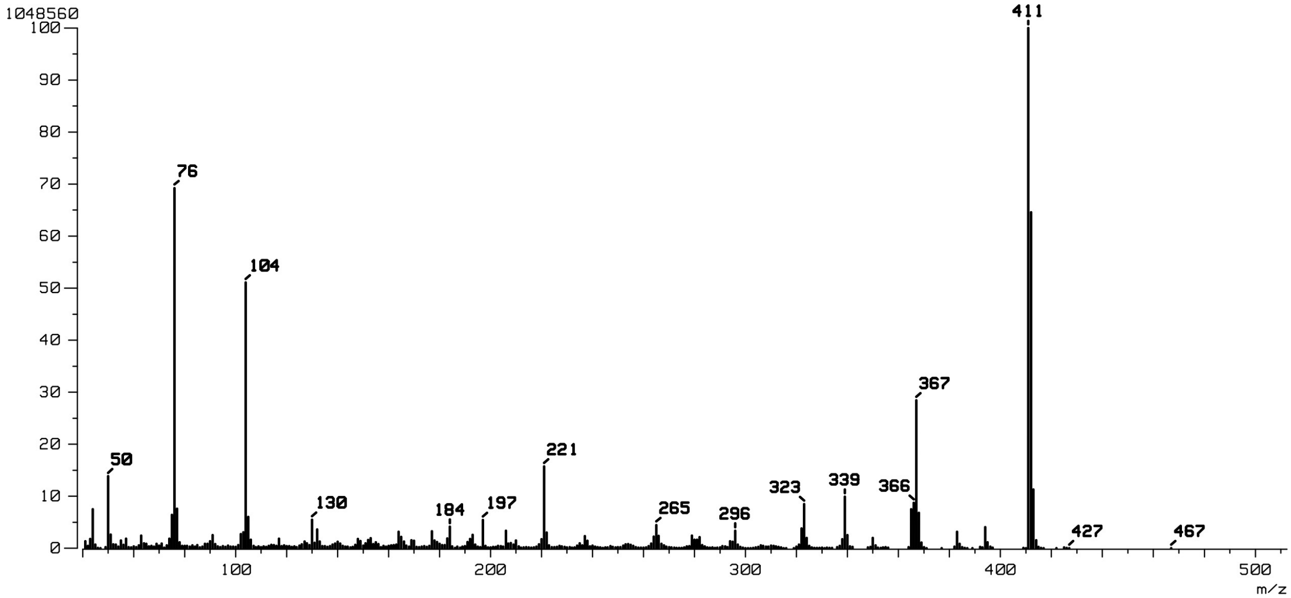

Figure S5: MS spectrum of 3,4-DITA.

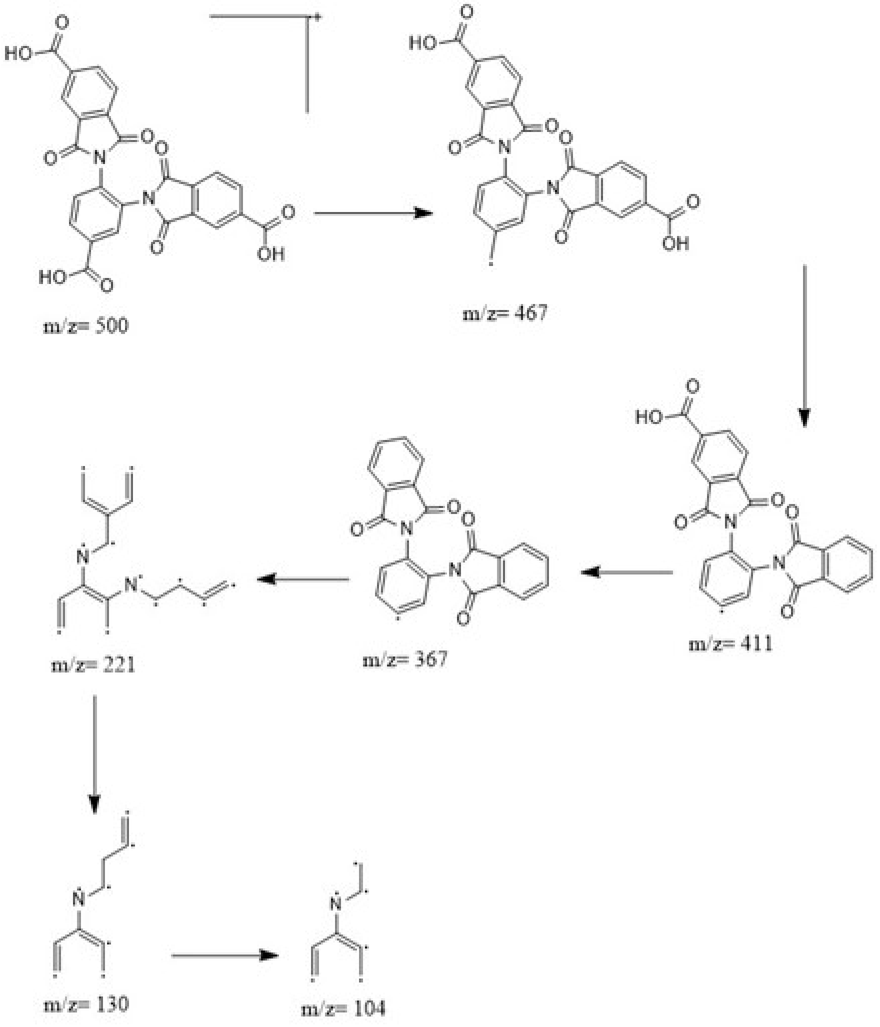

Figure S6: Fragmentation path of 3,4-DITA.

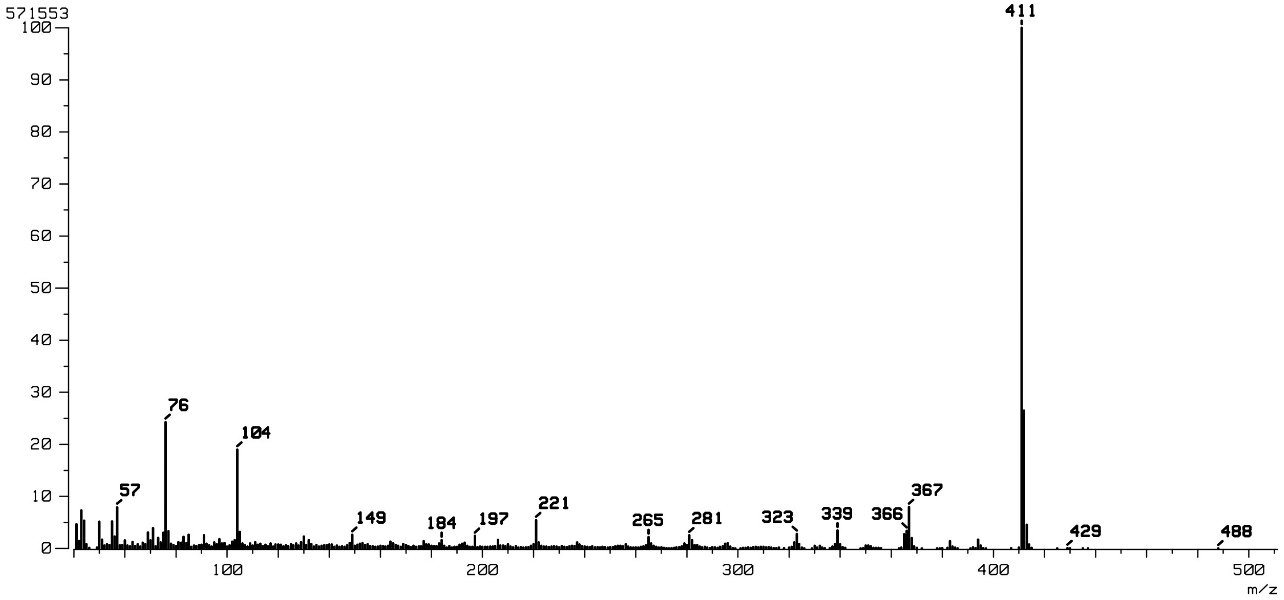

Figure S7: MS spectrum of 3,5-DITA.

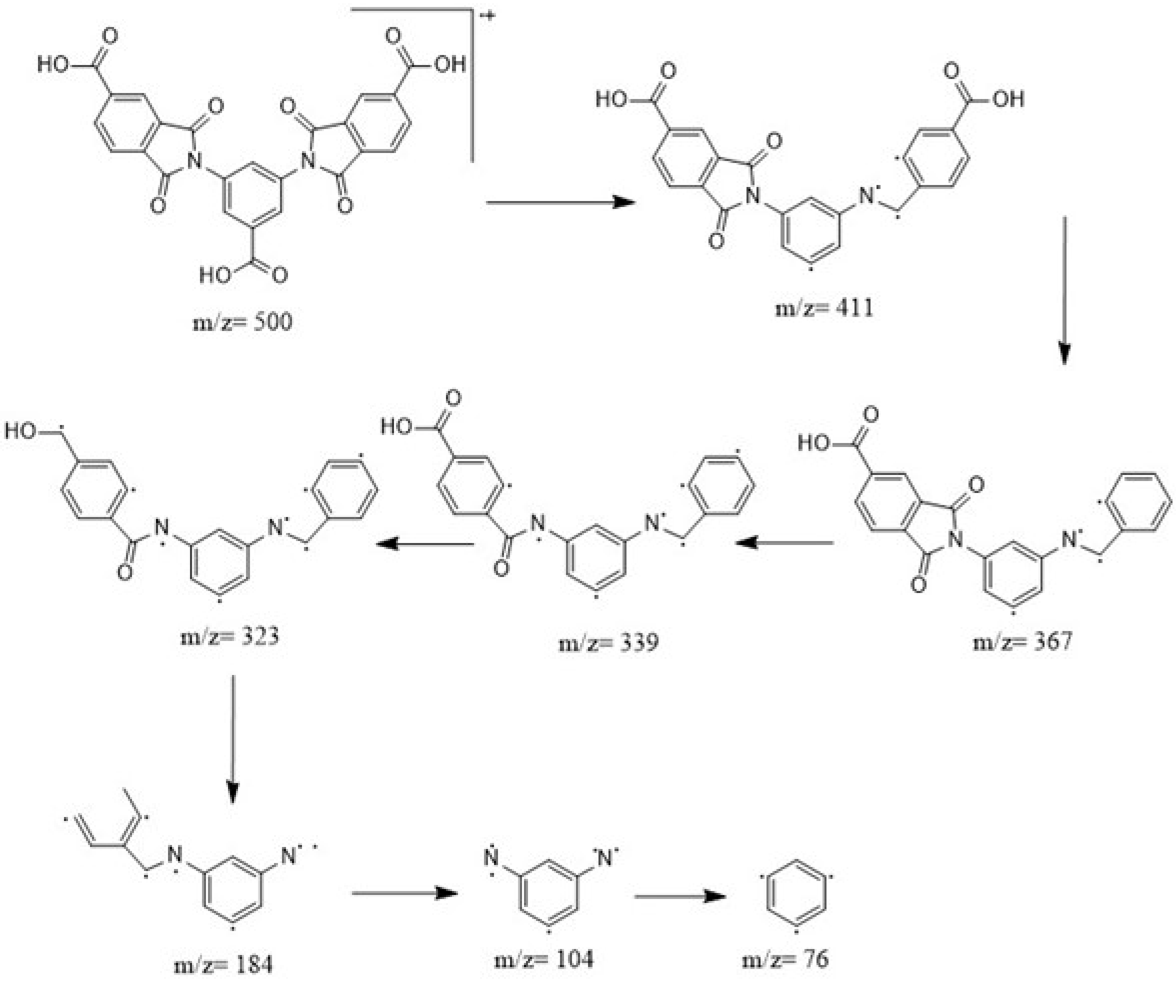

Figure S8: Fragmentation path of 3,5-DITA.

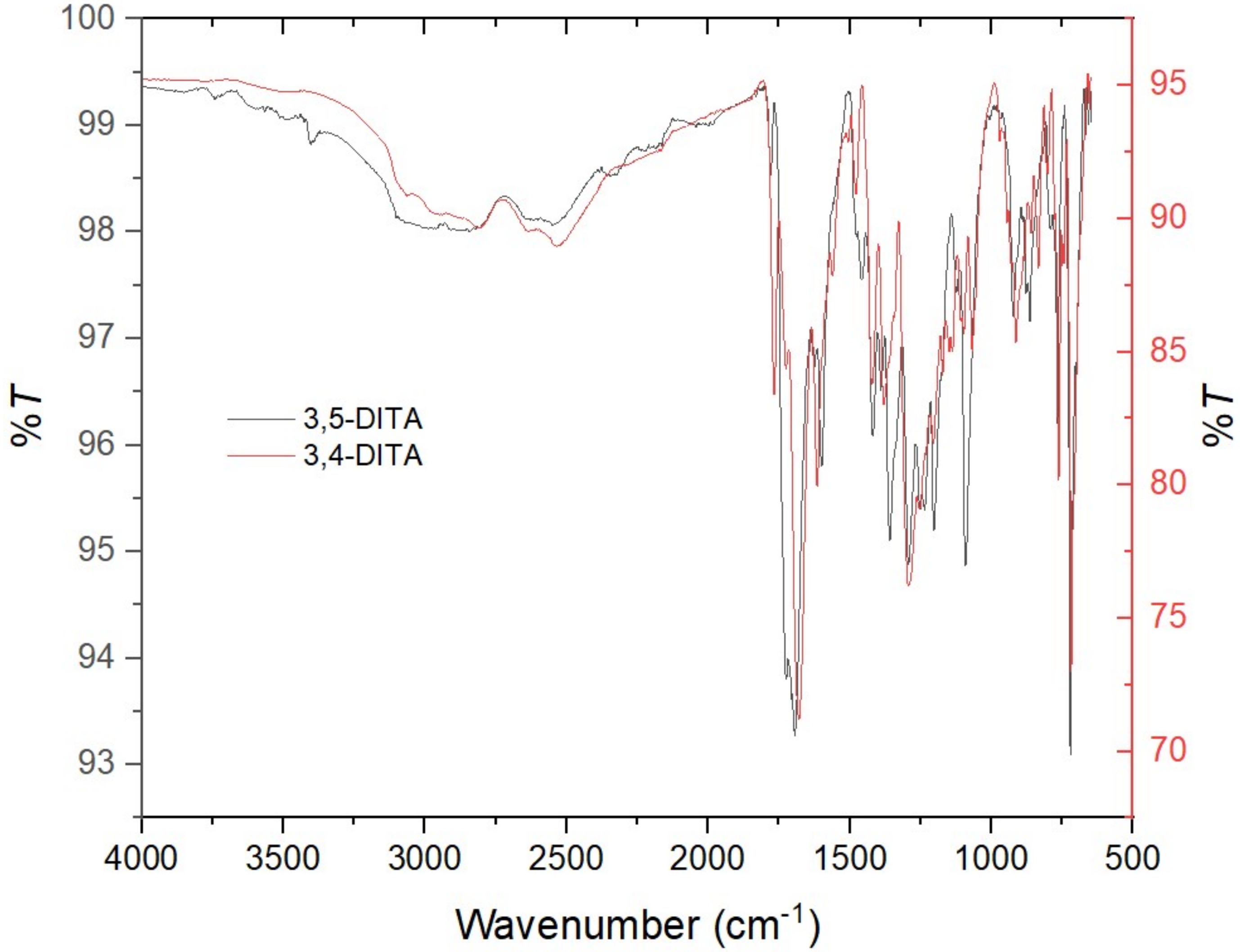

Figure S9: Comparison of FTIR of DITAs.

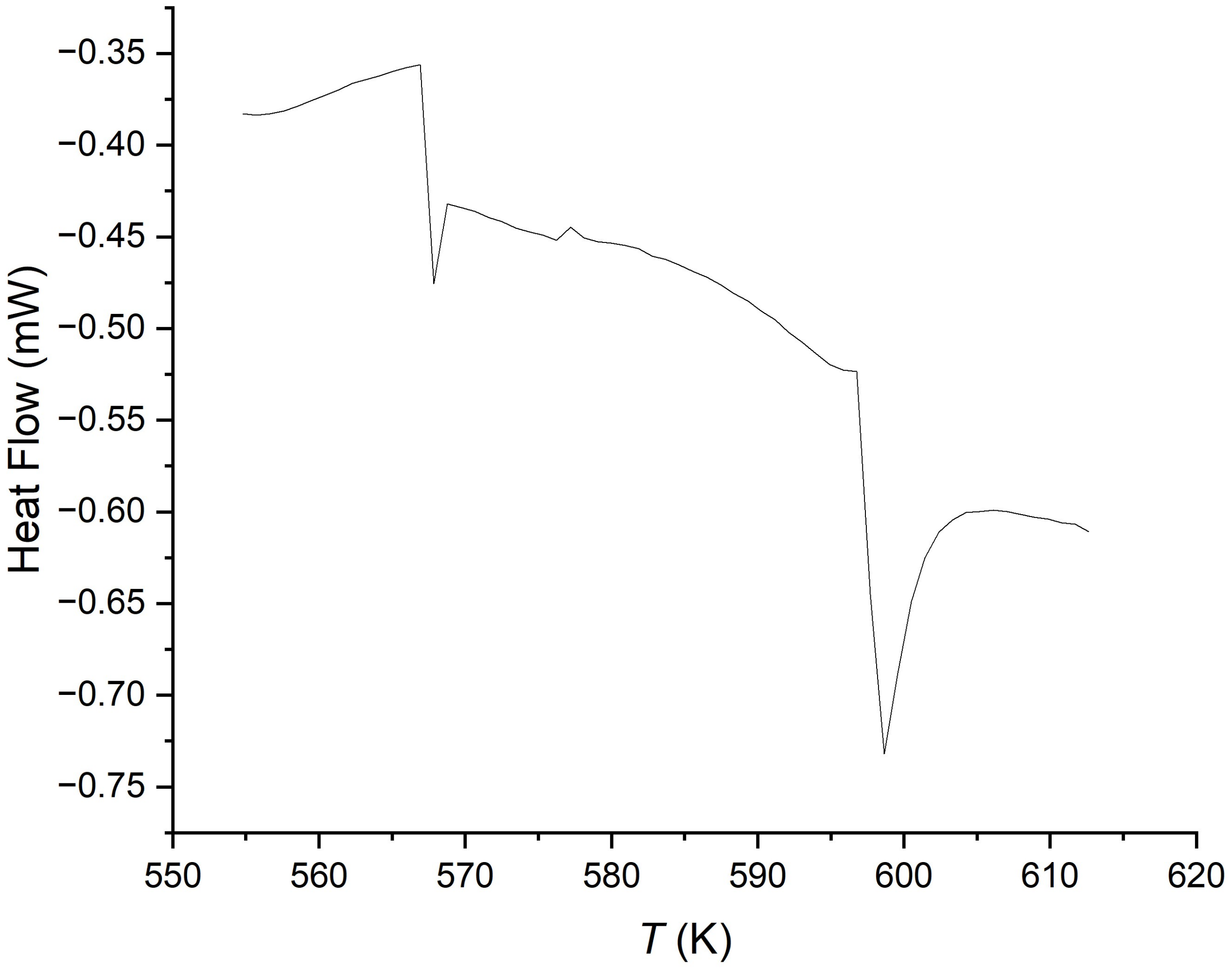

Figure S10: DSC thermogram of 3,4-DITA.

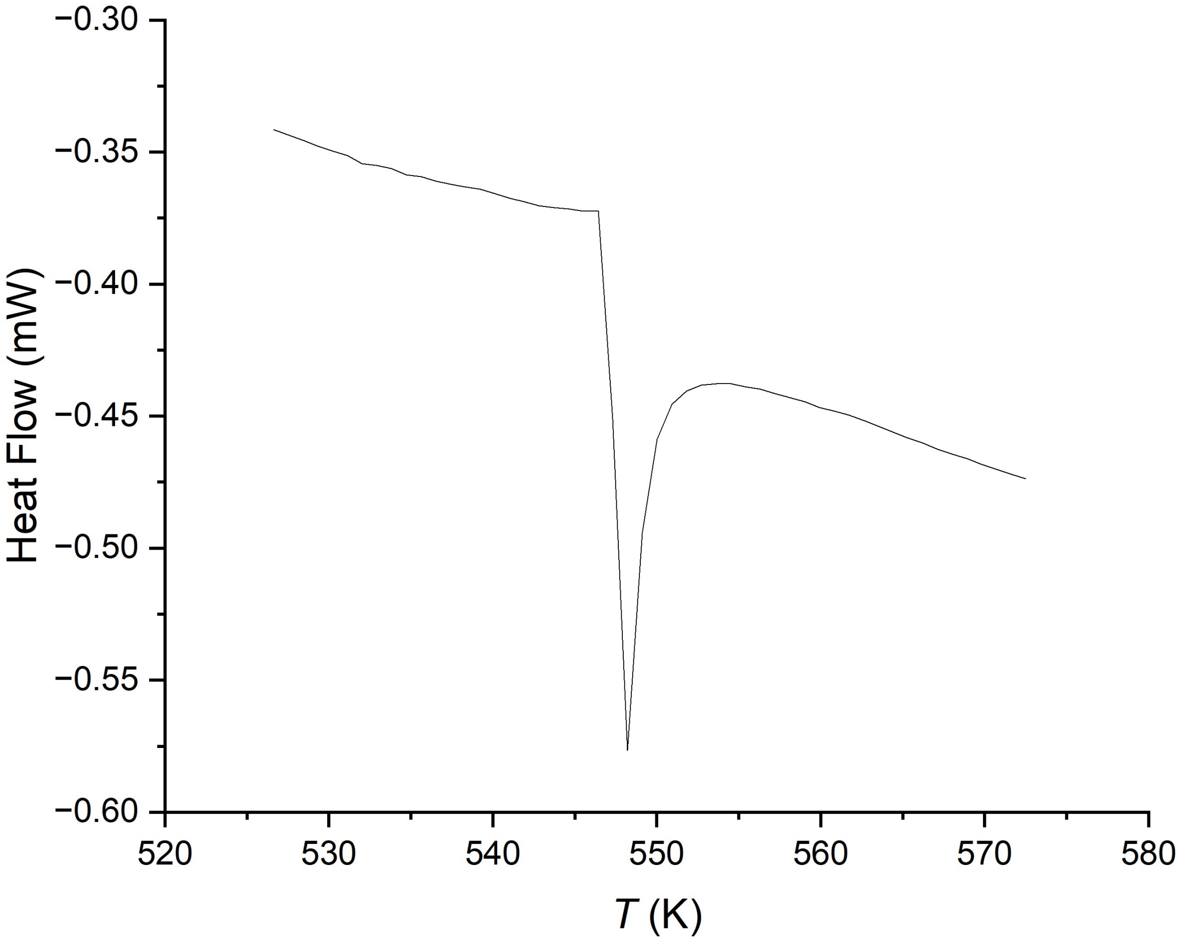

Figure S11: DSC thermogram of 3,5-DITA.

Table S1: Results for $C_{p,m}$(g) in J$\cdot$ mol$^{-1}\cdot$ K^{-1} of 3,4- and 3,5-DABA at different temperatures.

T/K	3,4-DABA	3,5-DABA	T/K	3,4-DABA	3,5-DABA
296	168.89	173.28	352	194.76	198.74
298.15	169.93	174.30	354	195.64	199.61
300	170.81	175.18	356	196.52	200.47
302	171.77	176.13	358	197.39	201.32
304	172.73	177.07	360	198.26	202.18
306	173.68	178.01	362	199.13	203.03
308	174.63	178.95	364	199.99	203.87

Continued on next page

Table S1 – continued from previous page

T/K	3,4-DABA	3,5-DABA	T/K	3,4-DABA	3,5-DABA
310	175.58	179.88	366	200.85	204.72
312	176.52	180.81	368	201.71	205.56
314	177.46	181.74	370	202.57	206.39
316	178.40	182.66	372	203.42	207.23
318	179.33	183.58	374	204.26	208.06
320	180.27	184.50	376	205.11	208.88
322	181.19	185.41	378	205.95	209.71
324	182.12	186.32	380	206.78	210.53
326	183.04	187.23	382	207.62	211.34
328	183.96	188.14	384	208.45	212.16
330	184.88	189.04	386	209.28	212.97
332	185.80	189.94	388	210.10	213.78
334	186.71	190.83	390	210.92	214.58
336	187.61	191.72	392	211.74	215.38
338	188.52	192.61	394	212.55	216.18
340	189.42	193.50	396	213.36	216.97
342	190.32	194.38	398	214.17	217.76
344	191.21	195.26	400	214.98	218.55
346	192.11	196.14	402	215.78	219.33
348	193.00	197.01	404	216.58	220.11
350	193.88	197.88			

Table S2: Results for $C_{p,m}$(g) in J· mol^{-1}· K^{-1} of 3,4- and 3,5-DITA at different temperatures.

T/K	3,4-DITA	3,5-DITA	T/K	3,4-DITA	3,5-DITA
296	479.36	479.99	456	668.55	668.90
298.15	482.26	482.89	462	674.51	674.85
300	484.75	485.37	468	680.38	680.71
306	492.77	493.38	474	686.18	686.50
312	500.74	501.33	480	691.89	692.21
318	508.63	509.21	486	697.52	697.83
324	516.46	517.02	492	703.08	703.38
330	524.21	524.76	498	708.55	708.85
336	531.90	532.43	504	713.95	714.24
342	539.50	540.03	510	719.27	719.56
348	547.03	547.54	516	724.52	724.80
354	554.49	554.98	522	729.69	729.96
360	561.86	562.35	528	734.78	735.05
366	569.15	569.63	534	739.81	740.07
372	576.36	576.83	540	744.76	745.02
378	583.49	583.95	546	749.64	749.89
384	590.54	590.99	552	754.45	754.69
390	597.50	597.94	558	759.19	759.43
396	604.38	604.81	564	763.86	764.10
402	611.18	611.60	570	768.46	768.70
408	617.89	618.30	576	773.00	773.23
414	624.52	624.92	582	777.48	777.70
420	631.06	631.45	588	781.89	782.10
426	637.52	637.90	594	786.23	786.45

Continued on next page

Table S2 – continued from previous page

T/K	3,4-DITA	3,5-DITA	T/K	3,4-DITA	3,5-DITA
432	643.89	644.27	600	790.52	790.73
438	650.18	650.55	602	791.93	792.14
444	656.39	656.75	604	793.34	793.54
450	662.51	662.86			

Table S3: Estimation of thermochemistry properties.

3,4-DITA
(3*CO-(O)(C_B))+(3*O-(H)(CO))+(7*C_B-(CO)(C_B)$_2$)+(9*C_B-(H)(C_B)$_2$)+(4*CO-N)(C_B)+ (2*C_B-(N)(C_B)$_2$)+(2*N-(CO)$_2$(C_B)+2*rsc+(3*radical 1)+(4*radical 2)+(1*Correction m-)+ (1*Correction p-)+H_{f0}<br>S°(g)=839.8 J mol^{-1}K^{-1}<br>$\Delta_f S^\circ$(g)=-1303.8 J mol^{-1}K^{-1}<br>$\Delta_f G^\circ$(g)=-1017.9 kJ mol^{-1}
3,5-DITA
(3*CO-(O)(C_B))+(3*O-(H)(CO))+(7*C_B-(CO)(C_B)$_2$)+(9*C_B-(H)(C_B)$_2$)+(4*CO-N)(C_B)+ (2*C_B-(N)(C_B)$_2$)+(2*N-(CO)$_2$(C_B)+2*rsc+(3*radical 1)+(5*radical 2)+(2*Correction m-)+ H_{f0}<br>S°(g)=848.0 J mol^{-1}K^{-1}<br>$\Delta_f S^\circ$(g)=-1295.6 J mol^{-1}K^{-1}<br>$\Delta_f G^\circ$(g)=-1023.8 kJ mol^{-1}

Table S4: Values calculated for the reaction of 3,4-DITA at different temperatures

T<br>K	K°	$\Delta_r G^{\circ}_m(g)$<br>kJ mol^{-1}	$\Delta_r H^{\circ}_m(g)$<br>kJ mol^{-1}	$\Delta_r S^{\circ}_m(g)$<br>J mol^{-1}K^{-1}
298.15	1.033E+12	-68.57	-82.86	-47.9
300.15	8.419E+11	-68.52	-82.45	-46.4
310.15	2.937E+11	-68.09	-80.42	-39.7
320.15	1.122E+11	-67.72	-78.34	-33.2
330.15	4.650E+10	-67.42	-76.23	-26.7
340.15	2.077E+10	-67.18	-74.07	-20.2
350.15	9.929E+09	-67.01	-71.86	-13.9
360.15	5.050E+09	-66.90	-69.62	-7.6
370.15	2.719E+09	-66.85	-67.34	-1.3
380.15	1.542E+09	-66.87	-65.01	4.9
390.15	9.179E+08	-66.94	-62.65	11.0
400.15	5.709E+08	-67.08	-60.24	17.1
410.15	3.699E+08	-67.27	-57.80	23.1
420.15	2.488E+08	-67.53	-55.31	29.1
430.15	1.734E+08	-67.85	-52.79	35.0
433.15	1.566E+08	-67.95	-52.02	36.8
440.15	1.248E+08	-68.22	-50.22	40.9

$U(\Delta_r H^{\circ}_m)$= 3 kJ· mol^{-1} and $U(\Delta_r G^{\circ}_m)$= 3 kJ· mol^{-1} which correspond to twice the combined uncertainty with a 95% confidence level.

Table S5: Values calculated for the reaction of 3,5-DITA at different temperatures

T<br>K	K°	$\Delta_r G^\circ_m(g)$<br>kJ mol^{-1}	$\Delta_r H^\circ_m(g)$<br>kJ mol^{-1}	$\Delta_r S^\circ_m(g)$<br>J mol^{-1}K^{-1}
298.15	2.090E+13	-76.03	-89.36	-44.72
300.15	1.641E+13	-75.93	-88.95	-43.37
310.15	5.264E+12	-75.53	-86.93	-36.75
320.15	1.858E+12	-75.20	-84.87	-30.21
330.15	7.153E+11	-74.92	-82.76	-23.75
340.15	2.979E+11	-74.72	-80.62	-17.35
350.15	1.333E+11	-74.572	-78.43	-11.0
360.15	6.372E+10	-74.491	-76.21	-4.77
370.15	3.234E+10	-74.472	-73.94	1.43
380.15	1.734E+10	-74.51	71.64	7.56
390.15	9.781E+09	-74.62	-69.29	13.64
400.15	5.782E+09	-74.78	-66.91	19.67
410.15	3.568E+09	-75.00	-64.49	25.63
420.15	2.292E+09	-75.29	-62.03	31.55
430.15	1.528E+09	-75.63	-59.53	37.42
433.15	1.362E+09	-75.74	-58.78	39.17
440.15	1.054E+09	-76.03	-57.00	43.23

$U(\Delta_r H^\circ_m)$= 2 kJ· mol^{-1} and $U(\Delta_r G^\circ_m)$= 2 kJ· mol^{-1} which correspond to twice the combined uncertainty with a 95% confidence level.
